# Improving Population Health Through Housing Policy: Lessons From the Public Housing Program

**DOI:** 10.1111/1468-0009.70098

**Published:** 2026-05-31

**Authors:** ANDREW FENELON

**Affiliations:** ^1^ Division of Epidemiology and Community Health University of Minnesota‐Twin Cities; ^2^ Minnesota Population Center University of Minnesota‐Twin Cities; ^3^ Life Course Center University of Minnesota‐Twin Cities

## Abstract

**Policy Points**

- Housing is a fundamental social determinant of health and is particularly amenable to policy intervention. Policies that seek to improve health should improve housing quality, affordability, and stability.
- The public housing program has improved health across the life course, but the structure of the program currently limits its applicability. Public housing can be adapted to foster policy innovation at the state level.
- A renewed public housing program should (1) serve a broader income spectrum; (2) integrate across levels of government; (3) capitalize on the social support benefits of project‐based housing; (4) emphasize effective administration; and (5) develop funding sources less vulnerable to political shifts.

**Context:**

Housing is a fundamental social determinant of health and is particularly amenable to policy intervention. However, the nationwide housing affordability crisis presents significant challenges for leveraging housing policy to improve population health in the United States. The US public housing program began during the New Deal, but has changed considerably since the mid‐20^th^ century. The program provides affordable and stable housing to nearly 1 million low‐income families, and may offer lessons for a way forward in improving health through better and more stable housing.

**Methods:**

Comparing the health impacts of US housing programs, public housing stands out as an unexpected success. Children in public housing experience better mental health, fewer emergency room visits, and greater housing stability. Adults report better physical and mental health, reduced risk of diabetes, and improved food security. However, recent federal housing policy has emphasized the tenant‐centered housing voucher program at the expense of public housing.

**Findings:**

While the contemporary public housing program improves health and well‐being across the life course, the early public housing program may offer lessons to improve outcomes in the 21^st^ century. Specifically, a renewed public housing could focus on high‐quality construction, emphasize effective management, and serve a broader spectrum of income levels. I also outline how public housing could benefit from an increasing role for state policy and enable integration across levels of government.

**Conclusions:**

Housing is particularly responsive to public housing, but current housing policies have proven insufficient to guarantee stable housing for all. The public housing program offers a promising way forward to leverage housing to address the US population health crisis.

Housing is so fundamental to conceptual frameworks of the social determinants of health that it would be difficult to describe the social determinants without explicitly referencing housing as a principal example.^1^ Indeed, the World Health Organization definition—“the conditions in which people are born, grow, live, work, and age”—explicitly references that “[p]eople who have limited access to quality housing … have a higher risk of illness and death.” Despite its theoretical and practical significance, housing remained an understudied factor in life course health and well‐being until the last 15 years.

In recent years, research on the effects of housing on health has grown in response to the expansion of data resources that collect information on both housing conditions and health outcomes. This research has also been facilitated by data linkages that combine information from multiple sources, often collected for different purposes. The best conceptual frameworks for housing and health recognize that housing has multifaceted effects on health through both direct and indirect mechanisms.^2^ Housing is a fundamental resource that allows individuals and families to focus on other areas of their lives, including improving their economic well‐being, family and social relationships, and health. When families face uncertainty surrounding the ability to afford safe and secure housing, health takes a back seat.^3^ The downstream impacts of housing insecurity can be catastrophic, leading to persistent material deprivation for decades, which shapes both physical and mental health.^4^, ^5^

The dimensions of housing that affect health are typically grouped into four categories: affordability, stability, quality, and neighborhood.^6^ Unaffordable housing—high housing costs relative to income—can strain household resources, drawing available funds away from health‐promoting inputs such as health care, healthy food, and enrichment.^7^ Unstable housing can precipitate disruptions to school and work, limit connections to social support, and impede access to care providers and effective disease management.^8^ Housing quality concerns can directly affect health through exposure to unsafe conditions, such as mold, lead, or crowded housing. Finally, housing is contextually situated, and neighborhood conditions can cumulatively affect health. While these dimensions of housing are often considered separately, they are inextricably linked to one another.^9^ For instance, families with constrained resources may opt to sacrifice on space, quality, or neighborhood to afford housing. Similarly, families may experience residential instability as they make multiple moves to remain able to afford their housing.^10^

Policy responses to the nationwide housing affordability crisis—such as housing vouchers—tend to rely on the private housing market to provide sufficient housing for low‐income families in the United States. However, I argue that public housing—the earliest federal rental housing assistance program—provides a promising avenue for future public investment in stable and affordable housing as a means to improve population health. Despite long‐standing disinvestment, the public housing program has enjoyed unexpected success in meeting housing needs for low‐income families, with evidence that it has multifaceted downstream benefits for health. The early US public housing program of the 1930s—which emphasized high‐quality construction and strong management–tenant relationships—offers insights about the potential to leverage the public provision of housing to improve US health in the future.

In this perspective, I argue that housing presents significant challenges for health policy but also offers several opportunities. As a social determinant of health, housing is particularly amenable to policy intervention, and policies that improve access to safe, stable, and affordable housing can have benefits in many different areas of individuals’ lives. While large‐scale housing supports have largely come from the federal government, there is an opening for policy innovation at the state level. I discuss the current evidence for leveraging housing policy to improve health. I then examine the state of the literature on the effects of rental assistance programs on health, highlighting an unexpected bright spot for the public housing program. Finally, I provide recommendations for a renewed focus on public housing as a means to improve health and reduce life course disparities. In doing so, I synthesize lessons from the early days of the public housing program in the 1930s as well as contemporary policy approaches to rental assistance in the United States.

## US Population Health Crisis

The United States currently finds itself in a population health crisis—life expectancy in the United States significantly lags that of other high‐income nations.^11^ While US life expectancy began to diverge from peer countries in the 1970s, the gap has grown more rapidly since 2010 and especially since the COVID‐19 pandemic.^12^, ^13^ Counterintuitively, these trends occur alongside both higher income levels as well as more rapid economic growth in the United States than in peer nations. Furthermore, American life expectancy varies substantially across geography, with some states seeing life expectancy similar to other high‐income countries and others having life expectancy similar to middle‐income countries.^14^, ^15^ However, US state differences also present a potential opportunity for public policy innovation, which has been shown to contribute to diverging life expectancy fortunes across states since at least 1980.^16^ To address the population health crisis, scholars encourage upstream policy innovation that leverages the social determinants of health,^17^ which presents both challenges and opportunities for housing policy.

## Challenges and Opportunities

The primary challenge of housing is that it is very costly and, for most families, is the largest expenditure. Furthermore, the nationwide housing affordability crisis exacerbates the challenge, as millions of families struggle to afford stable, good‐quality housing in a preferred location, with significant implications for US population health.^18^ More than 3 million families receive an eviction filing every year,^19^ and more than 40 million households experience housing affordability concerns, spending more than 30% of income on housing costs.^20^ Over time, housing costs have increasingly diverged from incomes in the bottom half of the income spectrum, such that it is not possible to afford a median market‐rate one‐bedroom apartment on a minimum‐wage income in any state.^21^ But while housing affordability concerns are most common and most acute among low‐income families (more than three‐fourths of families earning less than $30,000 a year pay too much for rent), inability to afford decent housing in a desirable location is common even among middle‐income families. Fully 45% of renter households with incomes from $40,000 to $75,000 spend more than 30% of income on rent.^20^

Another challenge is that housing's effects on health are multifaceted. At the most extreme manifestation of housing insecurity, homelessness represents a particularly important challenge. Rates of homelessness have risen rapidly in the pandemic era,^22^ and evidence suggests that individuals experiencing homelessness have higher rates of physical and mental illness, use fewer health care resources, and experience substantially elevated mortality compared with housed individuals.^23^, ^24^ Multiple aspects of housing insecurity impact health, including the inability to afford housing of adequate quality or size, making multiple moves, experiencing housing quality concerns, experiencing an eviction, and experiencing homelessness.^25^ Accounting for these varied dimensions of housing insecurity in research and addressing multiple dimensions through policy is not a simple task for policymakers.

Despite these challenges, housing is particularly amenable to public policy intervention, and it presents an opportunity to directly address the US population health crisis. Despite widespread evidence that housing affects health, it has largely resisted medicalization and remains squarely in the sphere of public policy, with myriad potential avenues for governments to support access to stable and affordable housing across the life course. These include direct supports such as funding the construction of publicly owned housing developments, subsidizing rent in private‐market units, or providing incentives for affordable housing development by private developers. Governments can also indirectly support housing development through zoning for more multifamily housing, particularly in high‐opportunity areas that have previously resisted it. In both cases, we have evidence that policy can be an effective means to promote better housing outcomes.^26^, ^27^, ^28^

Relatedly, housing policies can be implemented at many different levels of government. While rental assistance policies have largely been supported by the federal government through the US Department of Housing and Urban Development (HUD), state and municipal governments have also often developed their own programs to support access to affordable housing—such as Connecticut's Rental Assistance Program.^29^ In other cases, multiple levels of government partner to pool resources and expand the reach of housing programs, such as Minneapolis's Stable Homes Stable Schools program.

Finally, housing policy presents an opportunity to simultaneously improve outcomes in many domains of individuals’ lives. While improving the affordability and stability of shelter is an important goal in itself, the downstream benefits of better housing appear across a range of outcomes, including but not limited to physical and mental health, family stability, academic achievement, diet and physical activity, and voter turnout. And while housing exists squarely outside the health care system, improving housing can also improve outcomes relevant to the doctor–patient interaction, including chronic disease care and management.^30^, ^31^

## Current Rental Assistance Policies

Today, HUD operates rental assistance programs for low‐income families, providing affordable housing to 4.5 million families through three major programs: public housing, multifamily housing, and housing choice vouchers. Public housing refers to housing developments owned and operated by public housing authorities (PHAs), provided to assisted families at affordable rents.^32^ Multifamily housing programs involve subsidies to private development owners to maintain units at affordable rents for assisted tenants. Finally, the housing choice voucher program—formerly known as Section 8—provides families with a subsidy that allows them to enter the private rental market. The program seeks to provide flexibility in residential location, explicitly promoting mobility as a key focus. Since the 1990s, tenant‐based assistance through vouchers has increasingly defined HUD's orientation toward rental assistance at the expense of project‐based assistance through public housing.

These rental assistance programs focus on the most disadvantaged families.^33^ Although eligibility in theory includes families below 80% of area median income (AMI, typically referring to a metro area), in practice 75% to 80% of households receiving rental assistance have incomes less than 30% of AMI.^32^ Annual incomes for assisted families average around $18,000. The programs provide affordable housing by definition: families are expected to contribute 30% of income toward rent, with the difference covered either in‐kind via public housing or as part of a housing voucher.

Experimental and quasi‐experimental evidence suggests that rental assistance programs are effective at reducing homelessness, promoting housing stability, reducing crowding, and reducing exposure to poor‐quality housing.^34^, ^35^, ^36^, ^37^, ^38^ Unsurprisingly, there is also evidence that these benefits manifest in better health across the life course and into older age.^7^, ^39^, ^40^ However, evidence for health benefits tends to be strongest for public housing.

## The US Public Housing Program

The US public housing program developed during the New Deal intended to at once provide high‐quality rental housing for the growing urban working class and reinvigorate the struggling building trades during the Great Depression.^41^ It is the oldest rental assistance program in the United States and, until the 1990s, was the primary focus of low‐income rental housing policy.^42^ The earliest public housing developments, built in the 1930s and 1940s, were characterized by a high quality of construction, materials, and amenities that rivaled the quality of the best urban private housing of the era.^43^ The program was also not designed to serve only the poorest families. Instead, public housing was geared toward lower–middle‐income families who would benefit from improved housing but whose incomes were sufficient to afford rents in public housing. While rents tended to be lower than comparable housing in the private market, the magnitude of federal subsidies was not large enough to house the poorest families.^44^ The program represented a notable achievement in providing affordable access to high‐quality housing during a period in which at least one‐third of private rental housing was severely substandard.^45^

The public housing program of the 1930s and 1940s clearly differed in several ways from the contemporary program. In addition to emphasizing high‐quality construction and effective and well‐funded management, the program served both lower‐ and middle‐income families.^45^ As a result, rent revenue supported stronger operations and maintenance, which sustained the high quality of the early public housing developments.^43^ Beginning in the 1950s, public housing construction quality declined, owing to shifting federal priorities, including emphasis on suburban homeownership for middle‐class families. Additionally, federal regulations required PHAs to prioritize only the most disadvantaged households, which led to declining rent revenues, reduced operational and management capacity, and substantial deferred maintenance.^46^, ^47^ By the 1990s, the quality of many public housing developments had declined significantly, and federal housing policy became increasingly tenant‐centered, with the majority of rental assistance resources captured by the housing voucher program.

## An Unexpected Success

Popular knowledge of the benefits associated with public housing is often obscured by the program's reputation,^42^ which may reflect the long‐term reduction in federal resources for maintenance and operations. But despite decades of federal disinvestment, the evidence for positive health effects of contemporary public housing is strong. While health benefits of receiving rental assistance are occasionally observed regardless of the type of housing program, the most consistent evidence comes from public housing. Children whose families gain access to public housing experience more stable housing, are less likely to live in crowded housing conditions, and are less likely to experience rent burden.^37^, ^38^ These housing benefits also improve child and adult mental health,^39^, ^48^ reduce lead exposure,^49^ reduce food insecurity,^50^ diminish the risk of asthma‐related emergency department visits,^51^ and improve diabetes outcomes.^52^ Other studies find that effects are larger for public housing, such as for adult employment and earnings.^53^ Among older adults, public housing residents are less likely to report health problems and health care access challenges than those receiving other types of housing assistance.^54^ In contrast, findings for housing choice vouchers are much more mixed. An experimental study found that while housing vouchers significantly reduce housing cost burden and missed rent payments, they have few significant effects on child and adult well‐being. However, the study did show that vouchers reduce parental stress.^55^

The finding that public housing has disproportionate benefits runs in contrast to popular expectations about project housing and with HUD priorities surrounding housing assistance, but it is a notable regularity. One mechanism suggested to explain the unexpected success of public housing is the social support enabled by living in close‐knit housing developments. Qualitative evidence suggests that living in public housing can have benefits for access to informal social support, as tenants often draw on the resources of close‐knit networks within public housing developments.^56^ The value of these networks for health can be significant; individuals often have difficulty rebuilding their community after leaving public housing developments.^57^ Another potential mechanism is enhanced stability. Evidence suggests that public housing offers greater residential stability than other rental assistance programs,^37^ allowing tenants to maintain stronger connections to local institutions, develop relationships with health care providers, and devote time to disease management.^52^, ^58^ In contrast, the housing choice voucher program may reduce stability by encouraging residential moves to access new units. However, the difficulty of identifying landlords willing to accept vouchers may explain why only about 60% of voucher recipients successfully obtain a lease.^59^, ^60^

## Public Housing Challenges and Opportunities

Beginning in the 1950s, public housing entered a long‐term shift toward supporting only the most disadvantaged American families.^41^ Today, 75% of public housing tenants have incomes below 30% AMI. Since tenant rents are indexed to family income (30% of income paid toward rent), public housing rent revenue per tenant has declined over time, raising the per‐unit subsidy required for maintenance and operations. Because funding appropriations have not responded in‐kind to reductions in revenues from rent, this has in practice decreased the resources available for maintenance and effective management, in many cases weakening trust and collaboration between tenants and the housing authority. In addition to declining maintenance and deteriorating unit quality, increasing per‐unit subsidies have created downward pressure on the number of public housing units. The number of public housing units declined from 1.3 million in 1996 to 850,000 in 2025.^32^ Demand for rental assistance, however, remains high and greatly exceeds available units. Only around one in four eligible families receives assistance,^61^ and eligible families wait for an average of 27 months nationwide.^32^ Tenants who are waiting for assistance discuss the difficulties with navigating the housing bureaucracy alongside experiencing housing instability and, potentially, health challenges.^62^

These dynamics present a significant challenge for housing authorities to leverage the health and well‐being benefits of public housing, but also offer an opportunity for governments to expand the number of assisted families and increase revenues for maintenance and amenities. Specifically, there is an opportunity for state governments to invest in public housing that serves a broader spectrum of incomes and emphasizes effective management and residential stability.

## Recommendations for a Renewed Public Housing Program to Improve Population Health

Public programs that improve access to stable, affordable, high‐quality housing also improve health, and evidence supporting this is strongest for public housing. Despite the value of the program, federal housing policy has strongly focused on housing vouchers, which leverage the private rental market. Furthermore, it is likely that very little policy innovation on rental assistance will occur at the federal level, as policy activity for social programs has increasingly been concentrated at the state level.^63^ The early public housing program offers lessons for the future of housing policy, specifically for how states might leverage housing to improve health across the life course. These lessons are especially relevant in the resource‐constrained present, given the relatively modest financial subsidies provided for public housing during the 1930s.^44^ A renewed policy focus on housing and health should thus synthesize lessons from the early public housing program while leveraging the observed health benefits of the contemporary program:

### Serve a Broader Spectrum of Income Levels

The long‐term shift of public housing from supporting low‐ and moderate‐income families to serving only the most disadvantaged families negatively affected the effectiveness of the program. One of the historical challenges for housing assistance programs like public housing is that as the focus of the program shifted from the middle of the income spectrum to the bottom, the political power held by the program constituents also declined.^64^ As a result, the program lost necessary support from middle‐class families and became relegated to the whims of politicians looking to cut spending by reducing the welfare state.^65^ Producing housing for families above 50% AMI will have the dual benefit of reducing the concentration of disadvantaged families in public housing developments as well as expanding the political constituency for the program.^33^ As a bridge to increase rent revenue in public housing but avoid excluding the most disadvantaged tenants, Boston Housing Authority Administrator Kenzie Bok advocates for converting existing public housing developments to project‐based vouchers.^66^ This would enable market‐rate rent revenue for PHAs to support critical upgrades of public housing infrastructure, as in the ongoing modernization of Boston's St Botolph Apartments.

Expanding public rental housing to cover additional income levels would not only raise the economic diversity of public housing tenants but would also increase the total number of tenants that could be supported on the same absolute subsidy. It would also be an important step in expanding the role of states over the federal government.

### Enable Integration Across Multiple Levels of Government

Although rental housing supports have traditionally been the role of federal policy, state‐level policymaking has contributed significantly more to differences in life expectancy since the 1980s.^63^ One concern is that states do not command the same scale of resources as the federal government. That is, most states cannot afford to spend billions of dollars each year on housing for poor families at the scale required to effectively solve the problem. However, the early public housing program demonstrates that relatively modest subsidies for construction and management can provide high‐quality, stable housing at rents affordable to lower–middle‐ and middle‐income families. Furthermore, a state program could more effectively provide public housing in higher‐opportunity neighborhoods and integrate with other state‐administered programs such as food assistance.^67^

Currently, some states and localities also operate housing subsidy programs that complement the federal programs. For instance, the Connecticut Rental Assistance Program provides housing vouchers to 6,000 households, over and above the 78,000 supported by federal programs.^29^

### Capitalize on the Social Support Benefits of Project‐Based Housing

Research on the health benefits of public housing suggests that social support networks within project‐based housing developments may be an important mechanism.^68^ Contemporary rental assistance programs that emphasize residential mobility, such as housing choice vouchers, may overvalue the socioeconomic characteristics of tenants’ neighborhoods. In doing so, these programs may underrate the importance of residential and community stability for health and well‐being. A renewed investment in public housing should recognize the importance of these community ties for health and well‐being.^69^ The value of strong social ties for health also reflects the importance of housing stability, which is known to be a strength of public housing over housing vouchers. Stability is also a key determinant of access to health care and the management of chronic disease.

### Emphasize Effective Program Administration

Housing affordability is an important determinant of health,^70^ but it is important to pair affordable rents with effective and responsive management and maintenance. Early public housing emphasized strong relationships between housing authority management and tenants.^45^ Much of the criticism of public housing since the 1980s has centered on the perceived failure of housing authorities to complete timely maintenance, leading to examples of public housing developments in severe disrepair.^64^ However, as a public entity, the housing authority provides an avenue for tenant movements to improve conditions in public housing. Tenant organizers recognize the importance of having a landlord that is accountable to the public and can be pushed to respond to grievances.^71^ In contrast, the decentralized and market‐based nature of the voucher program may discourage accountability,^72^ and there is evidence that repairs tend to be delayed in voucher units.^73^

### Develop Funding Sources Less Susceptible to Political Shifts

Large‐scale financial support for housing in the United States occurs through several sources, and the type of support matters for the longevity of the policy. The federal government spends close to $60 billion annually on rental assistance programs. Financial support for homeownership is far higher, and often occurs in the form of forgone revenue—through tax‐free imputed rent or mortgage interest deduction—rather than through direct expenditures. As a result, policymakers must constantly defend rental assistance against portrayals as a welfare program, and recent attempts to expand the housing choice voucher program failed.^74^ In contrast, expenditures on homeowner supports are more likely to be perceived as the natural state of the housing market. To the extent possible, funding housing supports through forgone revenue in the tax code would make them less vulnerable to political pressure.

## Conclusion

Housing remains a major challenge for health policy, as the effects of unstable and unaffordable housing are felt across health domains, and current housing policies have proven insufficient to guarantee stable housing for all. However, housing is particularly responsive to public policy, and the field has ample evidence of housing policies that can improve health. As we move forward, it will be important to draw lessons from existing programs but also to recognize that policy innovation may largely occur at the state level. As increasingly important actors in policymaking, states have an opportunity to expand the reach of housing policy interventions to improve health and well‐being. At the same time, it is important to recognize that state housing policy development may very well expand the large disparities in health and mortality across states, as healthier states may be the first to improve their programs.^75^

Middle‐income American families increasingly face housing affordability concerns and struggle to find affordable housing in high‐cost cities with good job opportunities.^20^ A public option for housing represents a promising way forward as the housing affordability crisis has increasingly affected middle‐class families in addition to the poor. A renewed focus on public housing should combine the lessons of the early program—emphasis on quality, effective management, broad constituency—with the observed social and health benefits of the contemporary program. Synthesizing these approaches could lead to even larger improvements in population health while simultaneously allowing for integration between state and federal housing policy.

A renewed public housing program should not displace all other housing supports but should work synergistically with other programs, particularly those designed to respond to emergency situations. Evidence from pandemic‐era supports indicates that housing subsidies during 2020 and 2021 were effective at improving housing stability, reducing eviction, and improving well‐being. Pandemic‐era eviction moratoria were successful at keeping families housed during a period of economic precarity,^76^ and had wide‐ranging downstream benefits for health and mortality.^77^, ^78^, ^79^ Additionally, emergency rental assistance payments during the pandemic era—rent subsidies funded by the Coronavirus Aid, Relief, and Economic Security and American Rescue Plan Acts—were effective at reducing eviction, improving financial stability, and decreasing reports of anxiety.^80^, ^81^

Addressing the housing crisis is urgent, not least of all because it coincides with the American population health crisis. While direct evidence linking the underperformance of US life expectancy to housing is limited, these two crises developed during the same timeframe. The period in which US life expectancy deteriorated overlaps closely with the emergence of the housing crisis. To the extent that these trends are linked—or merely separate outcomes of an underlying trend—there is an opportunity to intervene. Policymakers should center stable, affordable, good‐quality housing with the recognition that the benefits will occur across many domains and for many years in the future.

## Funding/Support

The author received funding support from the Minnesota Population Center (P2CHD041023) and the Life Course Center (P30AG066613) at the University of Minnesota‐Twin Cities.

## Conflict of Interest Disclosures

The author has no conflict of interest.
